# Computing Pressure-Deformation Maps for Braided Continuum Robots

**DOI:** 10.3389/frobt.2019.00004

**Published:** 2019-02-05

**Authors:** David Navarro-Alarcon, Omar Zahra, Christian Trejo, Ernesto Olguín-Díaz, Vicente Parra-Vega

**Affiliations:** ^1^Department of Mechanical Engineering, The Hong Kong Polytechnic University, Kowloon, Hong Kong; ^2^Robotics and Advanced Manufacturing Group, Center for Research and Advanced Studies of the National Polytechnic Institute Saltillo Unit, Mexico City, Mexico

**Keywords:** continuum robots, self-organizing maps, adaptive systems, sensorimotor models, neural networks

## Abstract

This paper presents a method for computing sensorimotor maps of braided continuum robots driven by pneumatic actuators. The method automatically creates a lattice-like representation of the sensorimotor map that preserves the topology of the input space by arranging its nodes into clusters of related data. Deformation trajectories can be simply represented with adjacent nodes whose values smoothly change along the lattice curve; this facilitates the computation of controls and the prediction of deformations in systems with unknown mechanical properties. The proposed model has an adaptive structure that can recalibrate to cope with changes in the mechanism or actuators. An experimental study with a robotic prototype is conducted to validate the proposed method.

## 1. Introduction

Rigid robotic manipulators have been thoroughly studied and implemented in several applications for more than five decades now (Nof, 1999). More recently, many roboticists have turned their attention to the design and control of manipulators whose mechanical structure is deformable, and therefore, can achieve multiple shapes. This new type of *continuum robots* have many potential applications in fields such as medical robotics (monitoring procedures with flexible endoscopes), industrial robotics (grasping parts with compliant grippers), bio-inspired robotics (generating natural motions with soft limbs), to name a few cases (Hughes et al., 2016; Laschi et al., 2016). When compared to its rigid robot counterpart, methods for analyzing, sensing, and controlling soft robots are still in its infancy.

Pneumatic power is a common actuation method for continuum robots that brings some useful properties such as inherent compliance, motion backdrivability, controllable expansion/contraction of segments, etc. (Marchese et al., 2015; Sadati et al., 2016). However, the nonlinear and dynamic behavior of pneumatically-driven components makes it difficult to derive a closed-form analytical expression relating the driving air pressures and the highly deformable configuration of the robot. This expression is needed to accurately model and control the deformations of a system. Currently, there is no widely adopted approach for computing such sensorimotor relation.

Previous works have addressed this problem (e.g., Trivedi et al., 2008) presents a detailed nonlinear model relating pressures and the deformations due to pneumatic forces; (Shapiro et al., 2011) derives a closed-form relation between the segment's bending angle (measured on a plane) and the driving pressure; (Falkenhahn et al., 2015) presents a lumped parameters model using the Euler-Lagrange formalism; (Marchese et al., 2016) presents a model for multi-segment soft manipulators and experimentally identifies its parameters; (Sadati et al., 2017) derives a pressure-deformation model based on the principle of virtual work. Note that computing these models requires precise knowledge of the robot's mechanical properties, which are hardly available in practice due to the complexity of the system.

The objective of this manuscript is to present a new approach for automatically computing the steady-state relation between pressures and deformations. The method is inspired by models in neuroscience. Its topographically ordered structure resembles the way areas in the brain (e.g., the somatosensory and motor cortex) are organized according to related functions (Kohonen, 2001). This method creates discrete configuration clusters that can be used for predicting deformations and computing controls. In contrast with fixed analytical models, the proposed computational model can continuously adapt its structure to cope for new data or changes in the mechanical conditions. To the best of our knowledge, this is the first time this approach has been used for characterizing pneumatic-driven braided continuum robots.

## 2. Methods

### 2.1. Modeling of Braided Continuum Robots

**Figure 1:**
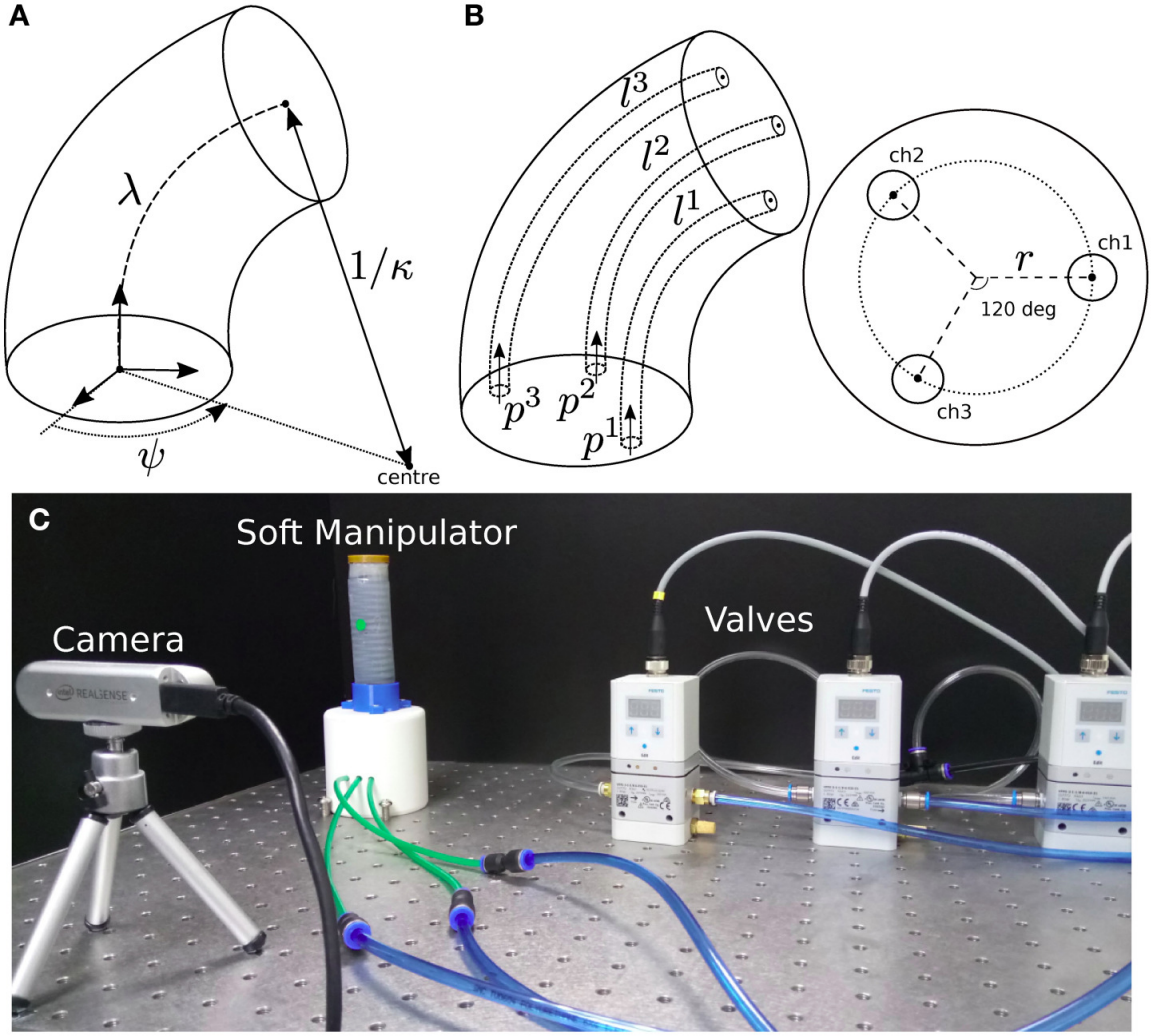
**(A)** Geometric representation of the robot's deformation coordinates **q** = [λ, κ, ψ]^⊺^. **(B)** Details of the internal pneumatic chambers to control the robot's configuration. **(C)** The experimental setup composed of an elastomer-based manipulator, pneumatic valves from Festo (VPPE-3-1-1), a RealSense camera from Intel, and a Linux-based PC with an i7-7700K processor.

Consider a cylindrical continuum robot (segment) with no radial and torsional deformations, and with constant curvature along its backbone. The configuration of this system is described by the deformation coordinates (Webster and Jones, 2010):

(1) $$\begin{array}{r} \text{q}={\left[\begin{array}{ccc} \lambda & \kappa & \psi \end{array}\right]}^{⊺}\in Q \end{array}$$

where λ denotes the length of the backbone, κ the curvature of the segment, and ψ the angle of the robot's bending (see Figure 1A). These deformation coordinates are determined by the lengths **l** = [*l*^1^, *l*^2^, *l*^3^]^⊺^ of the three pneumatic chambers. By using simple trigonometry, the relations between these two vectors can be obtained as follows (Sadati et al., 2017):

(2) $$\begin{array}{r} \text{l}=\left[\begin{array}{c} \lambda \left(1-\kappa r\cos\left(\psi \right)\right)\\ \lambda \left(1-\kappa r\cos\left(120^{{}^{\circ}}-\psi \right)\right)\\ \lambda \left(1-\kappa r\cos\left(120^{{}^{\circ}}+\psi \right)\right) \end{array}\right] \end{array}$$

where *r* denotes the backbone-chamber distance. The continuum robot is driven by three independent pressures **p** = [*p*^1^, *p*^2^, *p*^3^]^⊺^ ∈ P that are applied to its inner chambers (see Figure 1B). The dynamic equations of this system can be derived using Lagrangian-like methods (see e.g., Falkenhahn et al., 2015; Olguin-Diaz et al., 2018). To control the robot's shape, it is important to know what input pressures are needed to achieve a desired (final) deformation. This steady-state relation is characterized by the nonlinear mapping p=f(q):Q↦P, which can be found via the principle of virtual work (Hamill, 2014):

(3) $$\begin{array}{r} \delta \text{l}\cdot \left(\frac{\partial U^{b}}{\partial \text{l}}+\frac{\partial U^{e}}{\partial \text{l}}+\frac{\partial U^{d}}{\partial \text{l}}+\frac{\partial U^{p}}{\partial \text{l}}\right)=0 \end{array}$$

where *U*^*b*^, *U*^*e*^, *U*^*d*^, and *U*^*p*^ denote the potential energies resulting from the body loads, external loads, elastic deformations, and pneumatic pressures, respectively. Note that the fourth term above yields a function that must be solved for **p** (by exploiting Equation 2) to obtain the pressure-deformation relation. However, computing the above energy terms is a difficult task that requires the exact identification of the system.

### 2.2. Self-Organizing Configuration Maps

The complex properties of pneumatic continuum robots make it difficult to derive an expression relating pressures to deformations. In this work we show how an adaptive computational model can be used to approximate such nonlinear relation.

Consider first that the robot performs a series of babbling-like motions (Saegusa et al., 2009) around the workspace of interest described by P×Q. Let us assume that a set of *T* sampling points **p**(τ) and **q**(τ) are collected at the time instant τ and grouped into the following *training* data vector:

(4) $$\begin{array}{r} {\text{x}}^{k}=\left[\begin{array}{c} \text{p}\left(\tau \right)\\ \text{q}\left(\tau \right) \end{array}\right]\in P\times Q\;\text{for}\;k=1,\ldots ,T \end{array}$$

The vectors Equation(4) will be used for training a self-organizing map (SOM) (Kohonen, 2013). An important property of these maps is that they reduce the dimension of the input space into a 2D lattice while preserving its topology. Neighboring neurons represent configurations **x**^*k*^ that have “similar” pressure-deformation values.

The network has *N* computing units arranged in a 2D lattice. Each neuron has an associated weight vector **w**^*j*^ of the same dimension as **x**^*k*^. Training is done by sequentially presenting each input pattern **x**^*k*^ to the network and finding the weight vector that best matches its values. This “winning” neuron satisfies:

(5) $$\begin{array}{r} i=\underset{j}{\mathrm{argmin}}||{\text{w}}^{j}-{\text{x}}^{k}|| \end{array}$$

where *i* denotes its index over the *N* lattice nodes. The best matching neuron is placed at the center of a neighborhood of cooperating nodes. Let *h*^*ij*^ denote the neighborhood function centered at *i* for an active neuron *j*. To make the excitation proportional to the distance from the center, a common choice is to use the Gaussian function:

(6) $$\begin{array}{r} h^{ij}=\exp\left(-\frac{||{\text{r}}^{i}-{\text{r}}^{j}|{|}^{2}}{2\sigma_{t}^{2}}\right) \end{array}$$

where **r**^*i*^ and **r**^*j*^ denote the *i*th and *j*th node's 2D position within the lattice (e.g., **r**^*i*^ = [20, 15]). The variable σ_*t*_>0 specifies the effective cooperation width, i.e., the influence that neuron *i* exerts over the nearby neurons. The *j*th weight vector is computed with the following update rule (Sakamoto et al., 2004):

(7) $$\begin{array}{r} {\text{w}}_{t+1}^{j}={\text{w}}_{t}^{j}-\gamma_{t}h^{ij}\left({\text{w}}_{t}^{j}-{\text{x}}^{k}\right) \end{array}$$

for a learning gain γ_*t*_>0. By using Equation 6, the degree of adaptation of ${\text{w}}_{t}^{j}$ exponentially decreases with its separation from the center.

The variables σ_*t*_ and γ_*t*_ control the network's plasticity. These are first given large values to allow for coarse adaptations, and then decreased to fine tune the network's structure as follows:

(8) $$\begin{array}{r} \sigma_{t+1}=\sigma_{t}-\frac{1}{\eta }\left(\sigma_{t}-\tilde{\sigma }\right)\;\gamma_{t+1}=\gamma_{t}-\frac{1}{\eta }\left(\gamma_{t}-\tilde{\gamma }\right) \end{array}$$

for $\tilde{\sigma },\tilde{\gamma }>0$ as the fine tuning parameters, and η >0 as its time constant.

## 3. Results

### 3.1. Setup

We fabricated an elastomer-based soft manipulator (Agarwal et al., 2016; Schmitt et al., 2018) with three inner chambers that are independently controlled with pneumatic servo-valves (Festo) via an analog board (Phidgets). The robot's configuration is measured with a camera (see Figure 1C). All computations are performed in a Linux PC using OpenCV (Bradski, 2000).

In this study, we restrict our attention to the case of planar robot motions^1^. To produce plane deformations, we couple the controls *p*^2^ = *p*^3^ such that a virtual pressure separated by 180° from *p*^1^ is enforced. To differentiate between left/right bendings, we use signed curvature values and define a 2D deformation vector **q** = [λ, sgn(ψ)κ]^⊺^, where κ is measured with vision as in Navarro-Alarcon et al. (2014).

### 3.2. Experiments

The robot first performs a series of *slow* bending/stretching motions (by commanding ramp pressure signals to the valves) from which *T* = 734 data points **x**^*k*^ = [*p*^1^, *p*^2, 3^, λ, sgn(ψ)κ]^⊺^ are collected. This pressure/deformation data set is then used for training the network. The Supplementary Material Video S1 depicts the conducted experiments.

**Figure 2:**
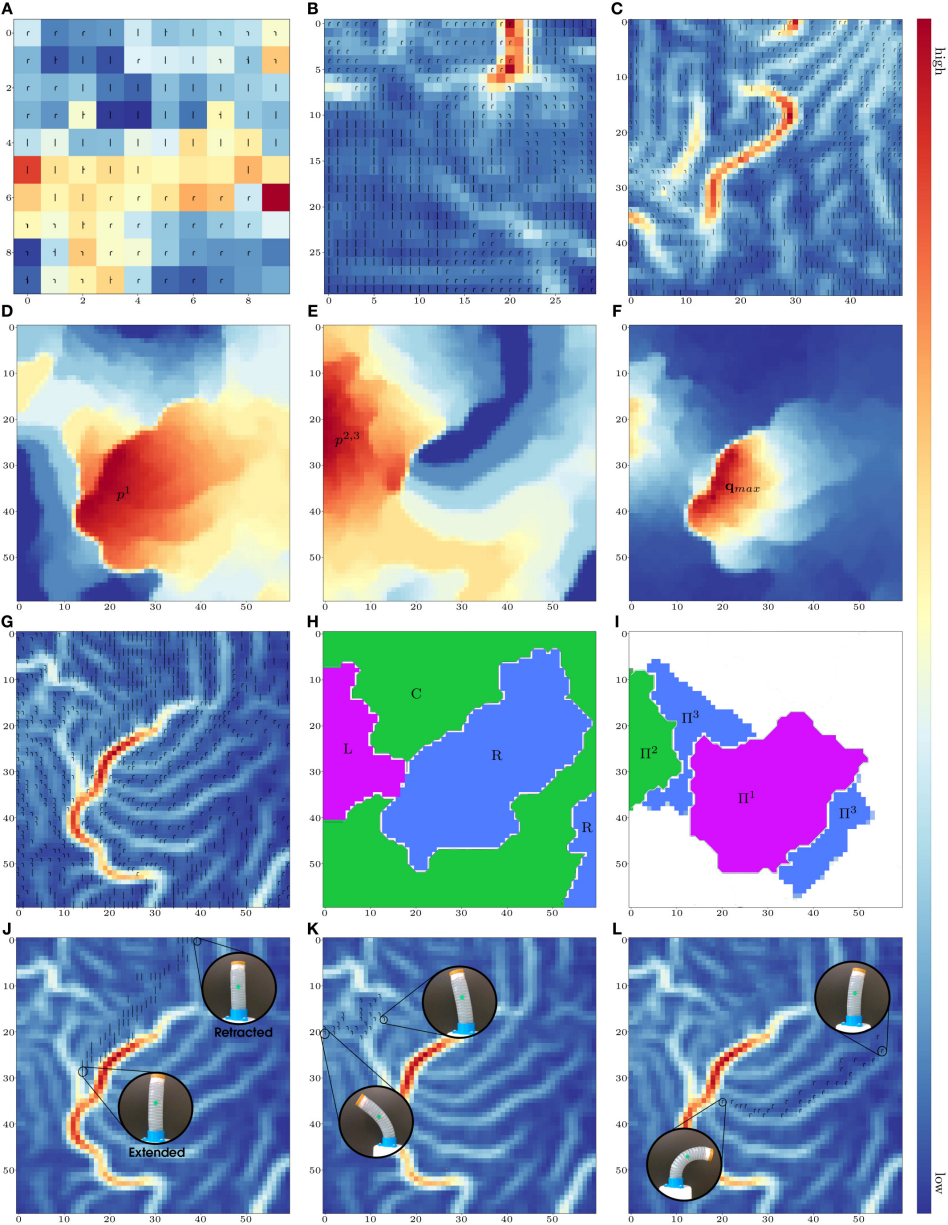
U-matrices with: **(A)** 10 × 10, **(B)** 30 × 30, and **(C)** 50 × 50 neurons. Magnitude of: **(D)** *w*^1*j*^ (activated with *p*^1^), **(E)** *w*^2*j*^ (activated with *p*^2, 3^), and **(F)** [*w*^3*j*^, *w*^4*j*^] (activated with **q**). **(G)** U-matrix with 60 × 60 neurons. **(H)** Deformation clusters. **(I)** Pressure clusters. Curves for **(J)** extension, **(K)** left bending, and **(L)** right bending.

Note that if the network has too few neurons, the model has problems in separating distinct configurations (e.g., left and right bendings). The U-matrix is a useful method to visualize boundaries between different configurations (Ultsch and Siemon, 1990). It assigns high/low values if its weight vector is different/similar from those of Neighboring nodes. Figures 2A-C depict the U-matrices obtained by considering lattices of 10 × 10, 30 × 30, and 50 × 50 neurons, respectively. These figures show that boundaries (i.e., red stripes) start to appear with a higher number of nodes^2^. For this study, we have selected a network with 60 × 60 neurons, which will be used for the results reported here.

Figures 2D,E depict the areas that are activated with the input pressures *p*^1^ and *p*^2, 3^. High values can be interpreted as deformations that are “mostly” enforced by either *p*^1^ or *p*^2, 3^ (i.e., right bending, and left bending). The map presents little overlapping of pressure regions; this corresponds to extension/retraction motions that require coordinated actions from all inputs. Figure 2F shows the group of neurons that encode the maximum value **q**_*max*_ for deformations (i.e., the longest and roundest configurations), which for our case corresponds to right bendings. The identified maximum region depends on the configurations available in the data set (in our experiments, left bendings were not so prominent).

Figure 2G shows the U-matrix computed for the 60 × 60 network with symbols denoting the dominant deformation state associated with each neuron. From this data, we can create clusters to represent the most numerically distinct configurations of the robot, namely, left and right large bendings, and central positions^3^. The resulting *deformation clusters* are shown in Figure 2H (where L, C, and R denote the left, center, and right robot configurations, respectively). We can also create the *pressure clusters* shown in Figure 2I, where Π^1^, Π^2^, and Π^3^ denote areas of “mostly” *p*^1^, “mostly” *p*^2, 3^, and combined pressure actions, respectively.

The network can be used to relate a target deformation $\bar{\text{q}}$ with its required pressure values $\bar{\text{p}}$. By presenting a partial vector **x^⊺^** = [^*^, ^**^, **q^⊺^**]^**⊺**^ (for ^*^ as unimportant terms) to the network, we can find the weight **w**^*j*^ that best matches $\bar{\text{q}}$, as done in Equation 5. The corresponding pressure values are simply located in the other coordinates of **w**^*j*^ = [*w*^1*j*^, *w*^2*j*^, ^*^, ^*^]^**⊺**^ ≈ [**p^⊺^**, ^*^, ^*^]^**⊺**^. Using this *prediction* approach, our trained network showed a maximum coordinate error of (3.57%, 5.38%) for the $\bar{\text{q}}$ and of (17.3%, 15.1%) for $\bar{\text{p}}$.

Figures 2J–L depict examples of how the topologically preserving network can capture incremental deformations of the robot into adjacent neurons. These figures show that the end points of the “deformation trajectories” correspond to the high/low values of the performed motion. The computed maps allow the characterization of the robot's properties, including fabrication errors, unbalanced pneumatic chambers (see the slight bending in Figure 2J), or any other variation in the sensorimotor conditions.

## 4. Discussion

This brief research report presents a computational model that approximates the steady-state pressure-deformation relations (as described by Equation 3) of pneumatic continuum robots. The method is based on a self-organizing network that discretises the configuration space while preserving its topology. This allows us to represent motions with contiguous nodes whose associated weights smoothly change along the trajectory. To evaluate the method's performance, we conducted experiments with a robotic prototype^4^.

The proposed method can be interpreted as an adaptive lookup table that automatically organizes data according to the similarity of its coordinates. Once the network is trained, each weight vector provides an approximated relation between deformations and pressures. Such relation can be re-trained when necessary (e.g., based on metrics Polzlbauer, 2004), by reinitializing the parameters in Equation 8 or by using dynamic SOMs (Rougier and Boniface, 2011).

There are some limitations with this approach, e.g., the accuracy of the sensorimotor model is directly dependant on the dimension of the network and the representativeness of the training data. Also, note that the computed model does not capture any dynamical properties of the mechanism; it only describes the sensorimotor relations in a static manner (see Sang et al., 2009 for an SOM to represent dynamical systems).

As future work, we plan to evaluate the method with 3D motions of a soft manipulator (using a 3D camera). To enable the use of sensor-based controls, we are currently developing a similar network that associates local Jacobian-like transformations to each node.

## Author Contributions

DN-A conceived the study, coordinated the project, and drafted the manuscript. OZ developed the adaptive algorithm, processed the data, and created the visualizations. CT fabricated the continuum robot and conducted the experiments. EO-D and VP-V developed the mathematical model and revised the paper.

### Conflict of Interest Statement

The authors declare that the research was conducted in the absence of any commercial or financial relationships that could be construed as a potential conflict of interest.

## Footnotes

^1^This situation removes 1-DOF from **q** as independent changes in ψ cannot be determined by its 2D image measurements.

^2^The left/right arc symbols are used for representing positive/negative curvatures, respectively, whereas the vertical line symbol is used for central configurations without prominent bendings.

^3^We used arbitrary threshold values to define these configurations.

^4^Note that if the data is collected from multiple (possibly heterogeneous) robots, the resulting map will average its properties.

## Supplementary Material

Video S1 Experiments.
